# A Vision-Language–Guided Multimodal Fusion Network for Glottic Carcinoma Early Diagnosis: Model Development and Validation Study

**DOI:** 10.2196/74902

**Published:** 2025-10-08

**Authors:** Zhaohui Jin, Yi Shuai, Yun Li, Mianmian Chen, Yumeng Liu, Wenbin Lei, Xiaomao Fan

**Affiliations:** 1 College of Big Data and Internet, Shenzhen Technology University, Pingshan District, 3002 Lantian Road, Shenzhen, Guangdong, 518118, China, 86 19276679344; 2 The First Affiliated Hospital, Sun Yat-sen University, Guangzhou, Guangdong, China

**Keywords:** glottic carcinoma early diagnosis, multimodal deep learning, large-scale foundation model, computer-aided diagnosis, clinical decision making

## Abstract

**Background:**

Early diagnosis and intervention in glottic carcinoma (GC) can significantly improve long-term prognosis. However, the accurate diagnosis of early GC is challenging due to its morphological similarity to vocal cord dysplasia, with the difficulty further exacerbated in medically underserved areas.

**Objective:**

This study aims to address the limitations of existing technologies by designing a vision-language multimodal model, providing a more efficient and accurate early diagnostic method for GC.

**Methods:**

The data used in this study were sourced from the information system of the First Affiliated Hospital of Sun Yat-sen University, comprising laryngoscopy reports and 5796 laryngoscopic images from 404 patients with glottic lesions. We propose a vision-language–guided multimodal fusion network (VLMF-Net) based on a large vision-language model for the early automated diagnosis of GC. The text processing module of this model uses the pretrained Large Language Model Meta AI (LLaMa) to generate text vector representations, while the image processing module uses a pretrained vision transformer to extract features from laryngoscopic images, achieving cross-modal alignment through the Q-Former module. By leveraging a feature fusion module, deep integration of text and image features is achieved, ultimately enabling classification diagnosis. To validate the model’s performance, the study selected contrastive language-image pretraining (CLIP), bootstrapping language-image pretraining with frozen image encoders and large language models (BLIP-2), a large-scale image and noisy-text embedding (ALIGN), and vision-and-language transformer (VILT) as baseline methods for experimental evaluation on the same dataset, with comprehensive performance assessment conducted using accuracy, recall, precision, *F*_1_-score, and area under the curve.

**Results:**

We found that on the internal test set, the VLMF-Net model significantly outperformed existing methods with an accuracy of 77.6% (CLIP: 70.5%; BLIP-2: 71.5%; ALIGN: 67.3%; and VILT: 64.3%), achieving a 6.1-percentage point improvement over the best baseline model (BLIP-2). On the external test set, our method also demonstrated robust performance, achieving an accuracy of 73.9%, which is 4.6 percentage points higher than the second-best model (BLIP-2: 69.3%). This indicates that our model surpasses these methods in the early diagnosis of GC and exhibits strong generalization ability and robustness.

**Conclusions:**

The proposed VLMF-Net model can be effectively used for the early diagnosis of GC, helping to address the challenges in its early detection.

## Introduction

Glottic carcinoma (GC) is a common malignant tumor of the head and neck [1]. According to GLOBOCAN, China reported 29,500 new cases and 16,900 deaths from laryngeal cancer in 2022 [2], posing heavy burdens on health care systems. Approximately 60% of patients are diagnosed at an advanced stage [3], leading to significant impairment of vital physiological functions and compromising both physical and mental health. Early diagnosis of malignant tumors has been increasingly emphasized in clinical practice due to its potential to improve cure rates and organ function preservation [4]. Therefore, optimizing diagnostic methods for GC and enhancing early detection capability are urgent tasks for otolaryngologists.

Laryngoscopy is the primary diagnostic tool for GC [1], offering direct visualization of lesion shape, extent, and surface texture. When combined with narrow band imaging, it enhances early tumor detection by identifying neovascularization [5], making it a valuable tool for early diagnosis. However, vocal cord dysplasia (VCD), a precancerous condition situated between normal epithelium and squamous cell carcinoma, is characterized by a small lesion with clinical and laryngoscopic features similar to early GC [6]. Therefore, it is challenging for the human eye to distinguish between them. In addition, the lesion is often covered by “leukoplakia-like” substance, which interferes with the ability of narrow band imaging to reveal submucosal vasculature [7], increasing the risk of misdiagnosis. Laryngoscopy reports provide textual descriptions of lesion morphology observed dynamically during the examination, supplementing static images and assisting in diagnostic decision-making. Studies have demonstrated a correlation between morphological grading and malignancy risk [8-10], underscoring the diagnostic value of textual reports. Furthermore, reports authored by experienced clinicians serve as valuable references, facilitating more accurate diagnoses by less experienced clinicians. Histopathology examination remains the gold standard for diagnosis [1]. However, biopsy is invasive, painful, and carries procedural risks [11], impeding its widespread application in large-scale clinical screening. To address this issue, it is necessary to develop an efficient and noninvasive method to improve the diagnostic accuracy of early GC.

Recently, significant progress has been made in deep learning techniques for tackling real-world classification tasks in computer vision and natural language processing [12-14]. Many researchers have sought to apply these models to the detection of laryngeal cancer, yielding promising outcomes. However, most existing methods only use laryngoscopic images as input, including UC-DenseNet [15], MTANet [16], Dlgnet [17], RedFormer [18], and SAM-FNet [19]. Although these methods have demonstrated promising performance in laryngeal cancer detection and other tasks, they neglect certain latent information present in other modalities. Such information is typically inaccessible through unimodal approaches, thereby highlighting the advantages of multimodal methodologies [20,21].

Therefore, this study proposes a novel method named vision-language–guided multimodal fusion network (VLMF-Net) for early diagnosis of GC. Addressing the limitations of traditional single-modality diagnostic methods, our approach integrates the images and reports text of laryngoscopy to provide a more comprehensive representation of lesion characteristics. Using a pretrained vision transformer (ViT) [22] model for image feature extraction and a LlaMa3 [23,24] model fine-tuned for text processing, we achieve effective multimodal feature fusion. Compared to single-modality methods, our approach significantly improves the diagnostic accuracy and robustness, achieving an accuracy of 0.776 on real clinical datasets. This study highlights the potential of multi-modal fusion in clinical auxiliary diagnosis and provides new insights for reducing misdiagnosis rates and improving patient treatment outcomes.

## Methods

### Dataset

In our study, we constructed 2 datasets for model development and validation. First, we built an internal dataset for model training, validation, and testing. For the internal dataset, we collected data from 404 patients with glottic lesions at the First Affiliated Hospital of Sun Yat-sen University in Guangzhou, China. This dataset consists of 5799 professionally annotated image-text pairs, covering two types of lesions: VCD and GC. Each sample includes a laryngoscopic diagnostic report written by an experienced otolaryngologist and its corresponding laryngoscopic image.

**Figure 1.**
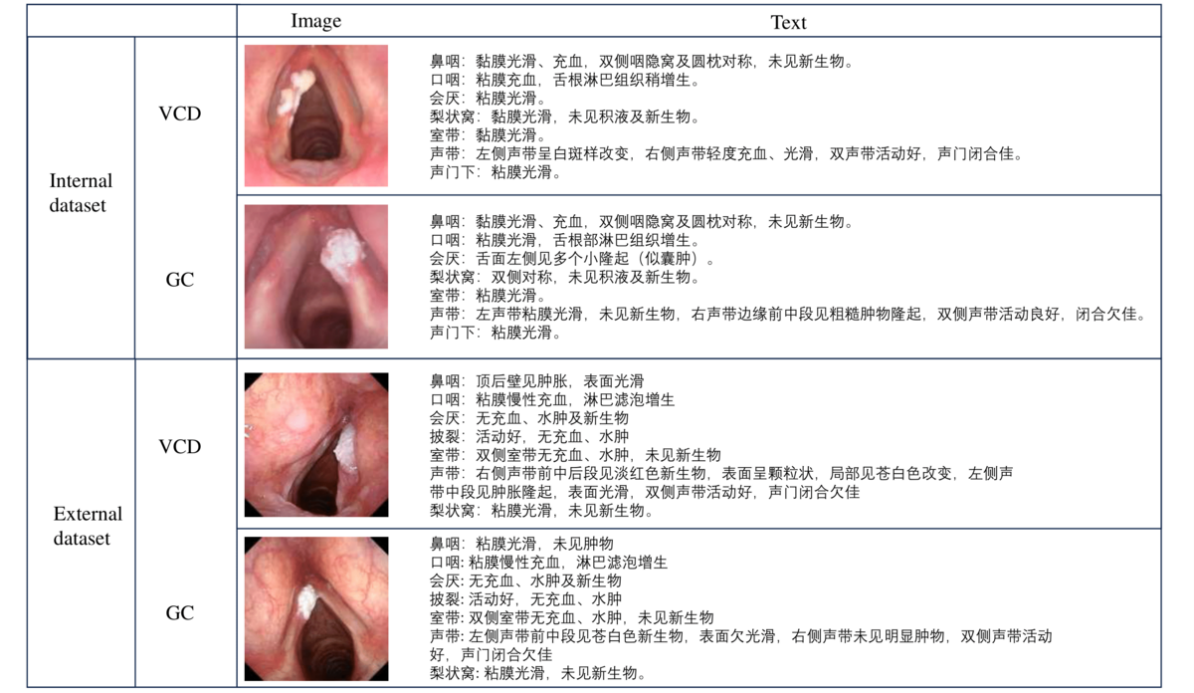
The specific form of data. GC: glottic carcinoma; VCD: vocal cord dysplasia.

In addition, to assess the model’s generalization ability and robustness, we constructed an external dataset. The external dataset was collected from the First People’s Hospital of Zhaoqing, consisting of data from 47 patients with glottic lesions between January 1, 2018, and August 31, 2024. This dataset includes 308 image-text pairs and strictly follows the principle of isolation from the training data, serving only for final performance evaluation. For detailed information on internal and external datasets, please refer to Figure 1 and Table 1.

**Table 1.** The statistics of datasets.

Datasets	Internal dataset	External dataset
Number of laryngoscopy reports	404	47
Number of VCD^a^	206	22
Number of GC^b^	198	25

a VCD: vocal cord dysplasia.

b GC: glottic carcinoma.

### Model Architecture

#### Overview

**Figure 2.**
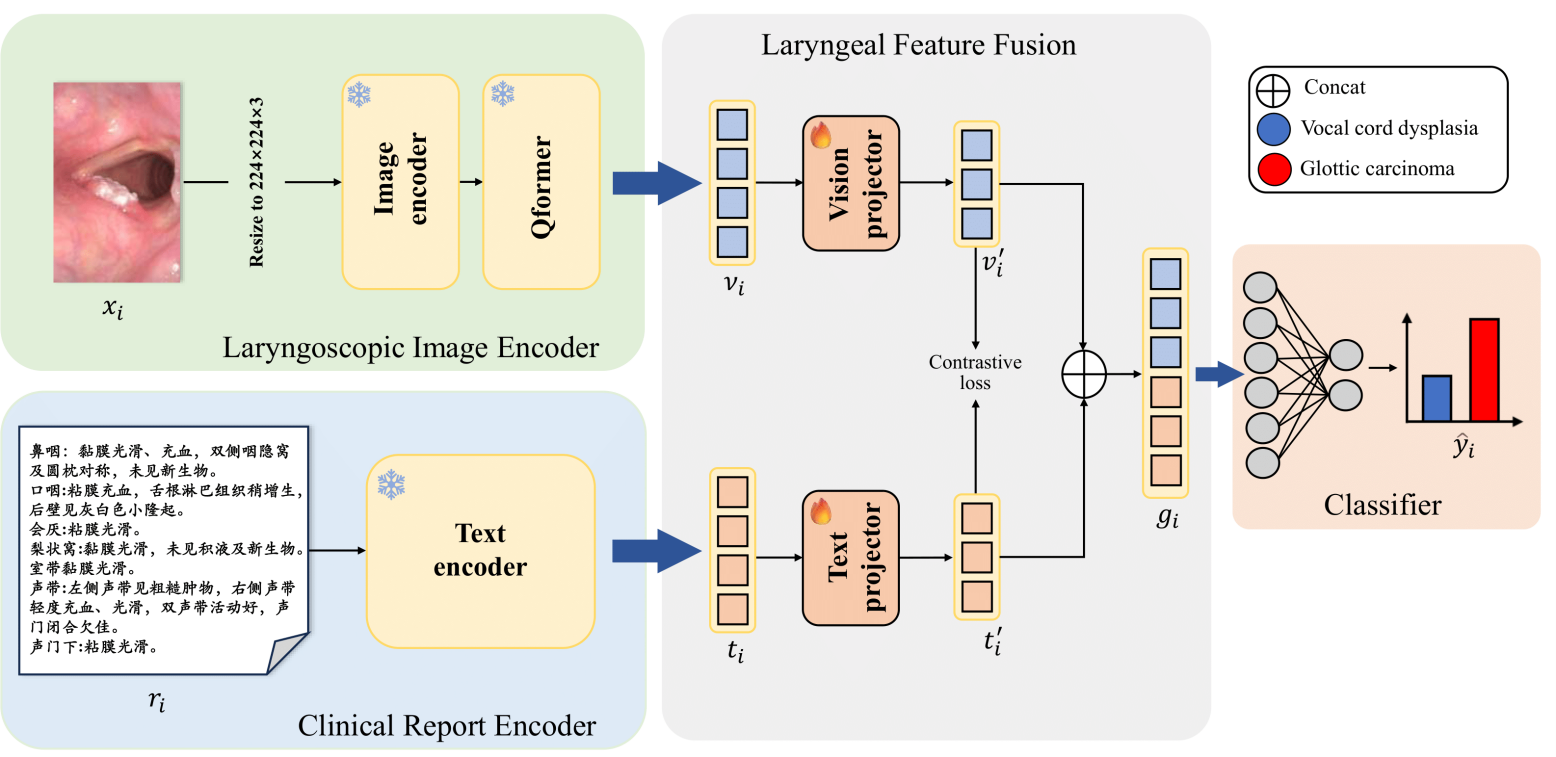
The overall architecture of our proposed vision-language–guided multimodal fusion network (VLMF-Net) model.

Figure 2 illustrates the overall architecture of our proposed VLMF-Net model, which consists of 3 main modules: the laryngoscopic image encoder, the clinical report encoder, and the laryngeal feature fusion module. Specifically, the laryngoscopic image encoder is responsible for extracting visual feature representations from laryngoscopic images, while the clinical report encoder captures textual feature representations from the patient’s laryngoscopic examination findings. These features are then fused through the laryngeal feature fusion module. Finally, the fused features are passed through a fully connected layer to complete the classification task. The detailed implementation of each module in our proposed VLMF-Net model is as follows:

#### Laryngoscopic Image Encoder

The laryngoscopic image encoder is an adapted version of a pretrained ViT [22] model. Considering that a single ViT model may introduce cross-modal discrepancies in multimodal tasks, potentially affecting model accuracy, we incorporated an additional Q-Former [25] module into the ViT model to mitigate the impact of modality differences and bridge the gap between image and text features. Q-Former is a trainable module based on Transformer that extracts and condenses visual features through alternating stacking of self-attention and cross-attention. Specifically, we first use ViT to extract features from the image, and then we input the extracted image features into a Q-Former module with frozen parameters to reduce modal differences.

Formally, let $f_{enc}(\cdot )$ denote the image encoder and $f_{q}(\cdot )$ denote the Q-Former module. Given an image $x_{i}$, first resize the image to 224×224×3, then the image feature $v_{i}$ is obtained as follows:


(1) $$v_{i}=f_{q}\left(f_{\text{enc}}\left(x_{i}\cdot \theta_{\text{enc}}\right);\theta_{q}\right)$$

where $\theta_{enc}$ and $\theta_{q}$ are the weight parameters of the image encoder and the Q-Former module, the shape of $v_{i}$ is 1×1024. It is worth noting that in terms of the weights of the pretrained model, we adopt the transfer learning strategy: we directly use the pretrained weights of the BLIP-2 [25] model as the weight parameters of the laryngoscope image encoder, because a large number of studies have demonstrated the effectiveness of BLIP-2 in downstream tasks [26-28]. Such operations can ensure that the extracted image features and text features are in the same scale space, thus effectively avoiding catastrophic problems caused by modal differences.

#### Clinical Report Encoder

In the clinical report encoder module, we use LLaMA3 [23], an advanced large language model, as the text feature extractor to obtain textual feature representations from the patient’s laryngoscopic examination findings. This model is renowned for its exceptional ability to understand long-form text.

Formally, let $f_{LLaMA3}(\cdot )$ denote the clinical report encoder function. Given a clinical report $r_{i},$ the process of obtaining the text feature $t_{i}$ can be formulated as follows:


(2) $$t_{i}=f_{\text{LLaMa3}}\left(r_{i};\theta_{q}\right)$$

where $\theta_{q}$represents the weight parameters of the clinical report encoder, the shape of $t_{i}$ is 1×4096. Notably, we adopt the same transfer learning strategy for the clinical report encoder’s weight parameters as we did for the laryngoscopic image encoder. Furthermore, considering that the text in our dataset is in Chinese, we use a fine-tuned LLaMA3 model trained on a Chinese dataset as the encoder’s weight source [24]. This approach effectively addresses the original LLaMA3 model’s limitations in Chinese language understanding.

#### Laryngeal Feature Fusion

In the 2 modules described above, we obtained image and text features. To effectively fuse these 2 modalities, we introduced the laryngeal feature fusion module, which aligns, maps, and integrates the features from both modalities. Specifically, we first use the vision projector $f_{vp}(\cdot )$ and the text projector $f_{tp}(\cdot )$ to map the image feature $v_{i}$ and text feature $t_{i}$ into a unified feature space:


(3) $$v_{i}^{\prime }=f_{\text{vp}}\left(v_{i};\theta_{\text{vp}}\right)$$

(4) $$t_{i}=f_{\text{tp}}\left(t_{i};\theta_{\text{tp}}\right)$$

Where $v_{i}^{`}$ and $t_{i}^{`}$ represent the mapped image and text features (their shapes are 1×512 and 1×2048), respectively, and $\theta_{vp}$ and $\theta_{tp}$ are the learnable parameters of the projectors $f_{vp}(\cdot )$ and $f_{tp}(\cdot )$ .

Next, we apply L2 normalization to both features to ensure consistency, obtaining:

$v_{i}^{``}=\frac{v_{i}^{`}}{||v_{i}^{`}{\left|\right|}_{2}}$and $t_{i}^{``}=\frac{t_{i}^{`}}{||t_{i}^{`}|{|}_{2}}$. Then$v_{i}^{``}$ and text feature$t_{i}^{``}$ along the feature dimension to form a vision-language joint representation, denoted as:$g_{i}=Concat\left(v_{i}^{``},t_{i}^{``}\right)$. This joint feature is then fed into a classifier, which consists of multiple fully connected layers, dropout layers, and ReLU activation functions. The classification process is formulated as:


(5) $${\hat{y}}_{i}=f_{\text{fc}}\left(g_{i},\theta_{\text{fc}}\right)$$

where $\theta_{fc}$ represents the learnable parameters of the classifier.

### Model Training

The internal dataset is divided at the patient level into training, validation, and test sets in a ratio of 8:1:1, strictly following the principle of data isolation. Regarding the loss function during training, we use the cross-entropy loss function $L_{ce}$ and the contrastive loss function$L_{ct},$ which are formulated as follows:


(6) $$L_{CE}=-\sum_{i=1}^{c}y_{i}\log({\hat{y}}_{i})$$

(7) $$L_{CT}=-\log\frac{\exp\left(\frac{sim(z_{i},z_{j})}{\tau }\right)}{\sum_{k=1}^{2N}1_{\left[k\neq i\right]}\exp\left(\frac{sim(z_{i},z_{k})}{\tau }\right)}$$

In $L_{ce}$, *C* represents the total number of classes, $y_{i}$ denotes the ground-truth class label in one-hot encoding, and $\hat{y_{i}}$ is the predicted probability for class *I*. This loss function aims to minimize the difference between the model’s predicted distribution and the true distribution, thereby guiding parameter updates and optimization. In $L_{ct}$, sim(·) represents cosine similarity,$z_{i}$ and $z_{j}$ are positive sample pairs, τ is the temperature parameter, and *N* is the number of negative samples. The final loss function $L_{loss}$ is formulated as follows:


(8) $$L_{loss}=L_{ce}+c\times L_{ct}$$

where c=0.1. All training processes and experiments are conducted on a dedicated server equipped with 4 NVIDIA A6000 GPUs with a total of 196GB of VRAM. The system runs on Ubuntu 20.04.5 LTS, and the model is implemented using PyTorch 3.9.0 and Scikit-learn 1.3.1. In this study, we use the AdamW optimizer to optimize VLMF-Net, with an initial learning rate set to 0.00001. A warm-up strategy and cosine learning rate scheduling are adopted to dynamically adjust the learning rate. VLMF-Net is trained for a total of 80 epochs.

### Ethical Considerations

This study was approved by the Ethics Committee of the First Affiliated Hospital of Sun Yat-sen University (approval number [2023]755‐1). Informed consent was waived by the institutional review boards of all participating hospitals due to the study’s retrospective design. We implemented stringent measures to protect the privacy of all participants by anonymizing all collected data to remove any personally identifiable information. Throughout the manuscript preparation, we diligently avoided disclosing any details that could reveal the identity of participants. Furthermore, no compensation or indemnity was required as the study did not cause any losses to participants beyond the necessary clinical diagnostic and therapeutic measures.

## Results

### Result of Our Model

**Figure 3.**
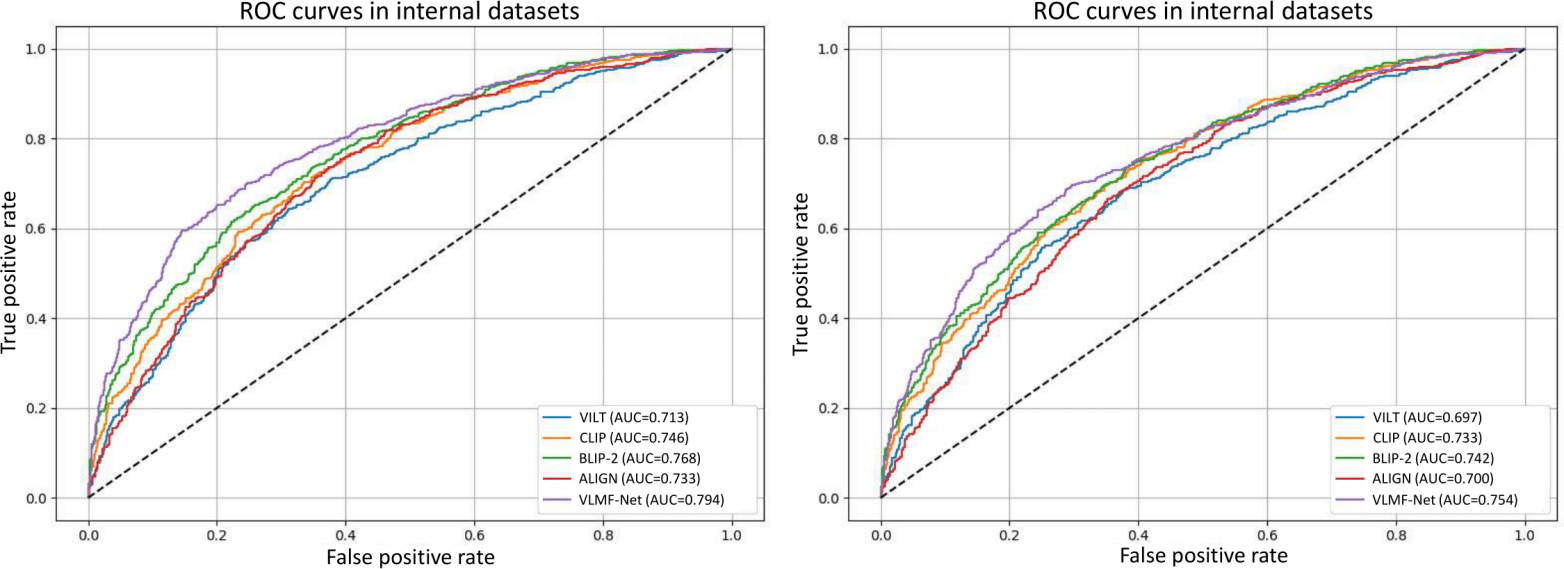
The ROC curves for different models. ALIGN: a large-scale image and noisy-text embedding; BLIP-2: bootstrapping language-image pretraining with frozen image encoders and large language models; CLIP: contrastive language-image pretraining; ROC: receiver operating characteristic; VILT: vision-and-language transformer; VLMF-Net: vision-language guided multimodal fusion network.

To demonstrate the effectiveness and advantages of our proposed VLMF-Net, we selected 4 classic models as baseline models and performed comparisons on 2 datasets. The 4 models include vision-and-language transformer (VILT) [29], contrastive language-image pretraining (CLIP) [30], bootstrapping language-image pretraining with frozen image encoders and large language models (BLIP-2) [25], and a large-scale image and noisy-text embedding (ALIGN) [31], and the 2 datasets refer to the internal and external datasets mentioned earlier. Tables2  3 present the average results of 5 trials for the 4 baseline models and our proposed model on the internal and external datasets, respectively. Figure 3 shows the receiver operating characteristic curves and corresponding area under the curve values of different models on internal and external datasets. As shown in Table 2, on the internal dataset, our method demonstrates significant advantages, achieving the following evaluation metrics: accuracy (0.776), precision (0.820), and *F*_1_-score (0.776). Notably, compared to the second-best model, our method achieves significant improvements of 0.061, 0.032, and 0.046 in accuracy, precision, and *F*_1_-score, respectively. In terms of class-wise recall, VLMF-Net achieves recall rates of 0.754 and 0.803 for VCD and GC, respectively. Compared to other models, VLMF-Net shows significant improvements across multiple evaluation metrics, indicating its superior ability in recognizing glottic cancer. In addition, as shown in Table 3, on the external dataset, our method also demonstrates significant advantages, achieving accuracy (0.739), precision (0.828), and *F*_1_-score (0.737), with improvements of 0.046, 0.053, and 0.046 over the second-best model, respectively. In terms of class-wise recall, our model achieves recall rates of 0.701 and 0.793 for VCD and GC, respectively. Compared to other models, there is also a significant improvement, which further demonstrates that VLMF-Net possesses excellent generalization ability and robustness. Moreover, as shown in Figure 3, our model demonstrates significant advantages in area under the curve metrics on both internal and external datasets. On the internal dataset, it outperforms the second-best model by 0.026, and on the external dataset by 0.012.

**Table 2.** Comparison with other multimodal models on an internal dataset.

Methods	Overall results			Recall of different classes	
	Accuracy, mean (SD)	Precision, mean (SD)	*F*_1_-score, mean (SD)	VCD^a^, mean (SD)	GC^b^, mean (SD)
VILT^c^	0.643 (0.02^d^)	0.677 (0.02^d^)	0.643 (0.01^d^)	0.632 (0.02^d^)	0.656 (0.01^d^)	
CLIP^e^	0.705 (0.01^d^)	0.788 (0.02^d^)	0.703 (0.01^d^)	0.672 (0.02^d^)	0.750 (0.01^d^)	
BLIP-2^f^	0.715 (0.02^d^)	0.770 (0.03^d^)	0.714 (0.02^d^)	0.694 (0.04^d^)	0.742 (0.03^d^)	
ALIGN^g^	0.673 (0.03^d^)	0.707 (0.02^d^)	0.673 (0.04^d^)	0.660 (0.02^d^)	0.688 (0.02^d^)	
VLMF-Net^h^	0.776 (0.01)	0.820 (0.02)	0.776 (0.01)	0.754 (0.02)	0.803 (0.01)	

a VCD: vocal cord dysplasia.

b GC: glottic carcinoma.

c VILT: vision-and-language transformer.

d *P*<.001.

e CLIP: contrastive language-image pretraining.

f BLIP-2: bootstrapping language-image pretraining with frozen image encoders and large language models.

g ALIGN: a large-scale image and noisy-text embedding.

h VLMF-Net: vision-language guided multimodal fusion network.

**Table 3.** Comparison with other multimodal models on an external dataset.

Methods	Overall results			Recall of different classes	
	Accuracy, mean (SD)	Precision, mean (SD)	*F*_1_-score, mean (SD)	VCD^a^, mean (SD)	GC^b^, mean (SD)
VILT^c^	0.631 (0.02^d^)	0.663 (0.02^d^)	0.630 (0.01^d^)	0.633 (0.01^d^)	0.628 (0.01^d^)	
CLIP^e^	0.686 (0.01^d^)	0.748 (0.02^d^)	0.685 (0.01^d^)	0.670 (0.02^d^)	0.708 (0.02^d^)	
BLIP-2^f^	0.693 (0.03^d^)	0.775 (0.02^d^)	0.691 (0.03^d^)	0.669 (0.03^d^)	0.726 (0.02^d^)	
ALIGN^g^	0.647 (0.02^d^)	0.680 (0.02^d^)	0.647 (0.03^d^)	0.642 (0.02^d^)	0.653 (0.03^d^)	
VLMF-Net^h^	0.739 (0.02)	0.828 (0.02)	0.737 (0.02)	0.701 (0.03)	0.793 (0.02)	

a VCD: vocal cord dysplasia.

b GC: glottic carcinoma.

c VILT: vision-and-language transformer.

d *P* value <.001.

e CLIP: contrastive language-image pretraining.

f BLIP-2: bootstrapping language-image pretraining with frozen image encoders and large language models.

g ALIGN: a large-scale image and noisy-text embedding.

h VLMF-Net: vision-language guided multimodal fusion network.

### Ablation Studies

To validate the effectiveness of our multimodal approach for early diagnosis of GC, we designed an ablation study to systematically evaluate the performance differences between models using single-modal and multimodal inputs: (1) M1: a single-modal model using only laryngoscopy images; (2) M2: a single-modal model using only laryngoscopy diagnostic reports; (3) M3: a multimodal model combining images and text, but without Q-Former. (4) M4: a multimodal model combining images and text with Q-Former. The experimental results, as shown in Table 4, indicate that the multimodal model M4 significantly outperforms both single-modal models across all evaluation metrics. Specifically, compared to the best single-modal model, M4 achieves improvements of 0.098 in accuracy (0.776), 0.106 in precision (0.820), 0.100 in recall (0.779), and 0.098 in *F*_1_-score (0.776). This demonstrates that the multimodal data fusion strategy effectively integrates visual features and textual semantic information, significantly enhancing the diagnostic performance of the model.

**Table 4.** Ablation study on vision-language–guided multimodal fusion network.

Variants	Image	Report	Q-Former	Accuracy, mean (SD)	Precision, mean (SD)	Recall, mean (SD)	*F*_1_-score, mean (SD)
M1	√	0.678 (0.02)	0.714 (0.03)	0.679 (0.03)	0.678 (0.03)
M2	√	0.673 (0.01)	0.711 (0.02)	0.675 (0.01)	0.673 (0.01)
M3	√	√	0.722 (0.02)	0.708 (0.01)	0.723 (0.01)	0.722 (0.03)
M4	√	√	√	0.776 (0.01)	0.820 (0.02)	0.779 (0.02)	0.776 (0.01)

In addition, to verify the effectiveness of the Q-Former module, we designed ablation experiments related to the Q-Former module. The experimental results are shown in Table 4. M4 outperforms M3 in all indicators. Specifically, M4’s accuracy (0.776) increased by 0.054, precision (0.820) increased by 0.112, recall (0.779) increased by 0.056, and *F*_1_-score (0.776) increased by 0.054. This indicates that the Q-Former module effectively reduces the impact of cross-modal differences.

### Visualization for Model Prediction

**Figure 4.**
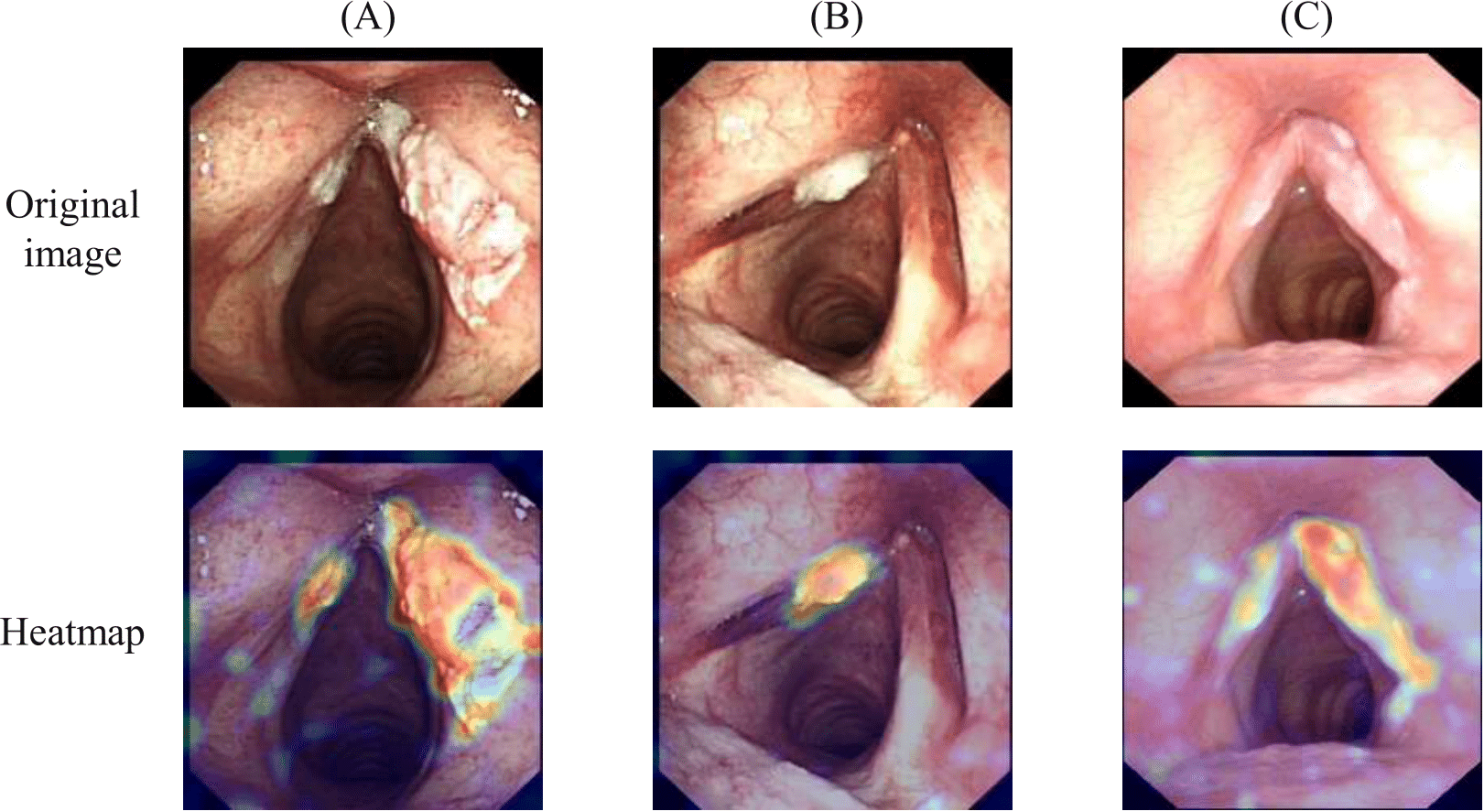
The heatmaps generated by the vision-language guided multimodal fusion network (VLMF-Net) model on patient laryngoscopic images, where (**A**) represents glottic carcinoma and (**B**) and (**C**) represent vocal cord dysplasia.

To enhance the interpretability of the VLMF-Net model’s predictions, we used the Grad-CAM (Gradient-Weighted Class Activation Mapping) [32] algorithm to generate class activation heatmaps. The visualization results are shown in Figure 4. In the heatmap, we can see that VLMF-Net focuses on the lesion area of the patient when analyzing the laryngoscopy image. This indicates that the model is able to correctly classify based on the features of the lesion area, enabling early diagnosis of GC.

## Discussion

### Main Findings

In our study, we developed a novel model for the early diagnosis of GC named VLMF-Net, which leverages multimodal fusion technology guided by vision-language information. When tested on an internal dataset, VLMF-Net achieved accuracy, precision, recall, and *F*_1_-score of 0.776, 0.820, 0.779, and 0.776, respectively, outperforming all baseline methods. These results demonstrate the feasibility and effectiveness of VLMF-Net in the early diagnosis of GC. Ablation studies further reveal that VLMF-Net significantly outperforms unimodal models. By integrating both laryngoscopic images and clinical text reports, VLMF-Net captures complementary diagnostic information, thus mitigating the risk of information loss inherent in single-modality systems. Notably, the textual modality meaningfully guides the extraction of image features; for instance, textual cues explicitly describing the lesion’s location (eg, “anterior-middle segment of the left vocal cord”) can help the model focus on the relevant visual regions. This form of cross-modal interaction may enhance the model’s ability to detect subtle pathological patterns that might otherwise be overlooked.

Moreover, Grad-CAM–based visualizations demonstrate that VLMF-Net consistently attends to clinically significant lesion areas. These attention heatmaps show strong alignment between the model’s focus and expert-defined pathological regions, enhancing interpretability. Such interpretability is crucial in clinical contexts, where transparency in decision-making processes fosters trust and facilitates integration of AI systems into diagnostic workflows.

Finally, these findings validate the design philosophy of VLMF-Net and highlight the broader potential of multimodal fusion strategies in medical AI. In particular, the ability to synthesize visual and contextual clinical information allows for more robust and informed diagnostic decisions. This work not only advances the state of the art in GC diagnosis but also lays a foundation for extending vision-language multimodal techniques to other complex diagnostic tasks where rich multimodal data is available.

### Deep Learning Challenges in Early GC Diagnosis

Early GC lesions are usually small and exhibit complex morphological characteristics, making it challenging for deep learning models to capture fine-grained lesion features. As a result, important details related to the lesions may be missed, significantly affecting the model’s diagnostic accuracy. Furthermore, most existing mainstream models rely solely on laryngoscopic images as input. While these models have achieved some progress in a unimodal setting, they overlook information from other modalities, which is often beyond the reach of image-based models [15-19]. This limitation constrains the model’s comprehensive understanding of lesion characteristics and ultimately affects diagnostic accuracy.

### Strengths and Limitations

To the best of our knowledge, this is the first study to apply multimodal techniques to the early diagnosis of GC. In our model, we fully utilize patients’ laryngoscopy reports and laryngoscopic images to extract relevant information about GC, enabling the model to comprehensively understand the patient’s condition and thereby improve its performance. However, this study still has some limitations. First, the VLMF-Net, based on a pretrained large language model, relies heavily on powerful computational resources, particularly high-performance GPUs, during training, which may result in slower training and inference speeds. Second, the data used in this study underwent quality checks, but in real-world scenarios, more complex situations may arise, such as poor image clarity due to imaging devices or challenging shooting angles, which could affect the model’s diagnostic accuracy.

### Conclusions

In this paper, we propose a VLMF-Net for the early diagnosis of GC. Extensive experiments on 2 datasets demonstrate that VLMF-Net achieves superior accuracy and robustness, effectively addressing the challenges of early GC diagnosis.

## Abbreviations

ALIGN: a large-scale image and noisy-text embedding
BLIP-2: bootstrapping language-image pretraining with frozen image encoders and large language models
CLIP: contrastive language-image pretraining
GC: glottic carcinoma
Grad-CAM: Gradient-Weighted Class Activation Mapping
LLaMa: Large Language Model Meta AI
VCD: vocal cord dysplasia
ViT: vision transformer
VLIT: vision-and-language transformer
VLMF-Net: vision-language guided multimodal fusion network
